# Molecular Epidemiology of *Fonsecaea* Species

**DOI:** 10.3201/1703.100555

**Published:** 2011-03

**Authors:** Mohammad Javad Najafzadeh, Jiufeng Sun, Vania A. Vicente, Corne H.W. Klaassen, Alexandro Bonifaz, A.H.G. Gerrits van den Ende, Steph B.J. Menken, G. Sybren de Hoog

**Affiliations:** Author affiliations: Centraalbureau voor Schimmelcultures Fungal Biodiversity Centre, Utrecht, the Netherlands (M.J. Najafzadeh, A.H.G. Gerrits van den Ende, G.S. de Hoog);; University of Amsterdam, Amsterdam, the Netherlands (M.J. Najafzadeh, S.B.J. Menken, G.S. de Hoog);; Mashhad University of Medical Sciences, Mashhad, Iran (M.J. Najafzadeh);; Sun Yat-Sen University, Guangzhou, Guangdong, People’s Republic of China (J. Sun, G.S. de Hoog);; Federal University of Paraná, Curitiba, Paraná, Brazil (V.A. Vicente);; Canisius Wilhelmina Hospital, Nijmegen, the Netherlands (C.H.W. Klaassen);; Hospital General de México, Narvarte, Mexico (A. Bonifaz);; Research Center for Medical Mycology, Beijing, People’s Republic of China (G.S. de Hoog)

**Keywords:** Chromoblastomycosis, Fonsecaea, distribution, amplified fragment-length polymorphism, AFLP, fungi, research

## Abstract

To assess population diversities among 81 strains of fungi in the genus *Fonsecaea* that had been identified down to species level, we applied amplified fragment-length polymorphism (AFLP) technology and sequenced the internal transcribed spacer regions and the partial cell division cycle, β-tubulin, and actin genes. Many species of the genus *Fonsecaea* cause human chromoblastomycosis. Strains originated from a global sampling of clinical and environmental sources in the Western Hemisphere, Asia, Africa, and Europe. According to AFLP fingerprinting, *Fonsecaea* isolates clustered in 5 groups corresponding with *F. pedrosoi*, *F. monophora*, and *F. nubica*: the latter 2 species each comprised 2 groups, and *F. pedrosoi* appeared to be of monophyletic origin. *F. pedrosoi* was found nearly exclusively in Central and South America. *F. monophora* and *F. nubica* were distributed worldwide, but both showed substantial geographic structuring. Clinical cases outside areas where *Fonsecaea* is endemic were probably distributed by human migration.

## Abstract 2

These fungi disperse slowly, leading to changes in structure at different geographic locations.

The genus *Fonsecaea* comprises etiologic fungal agents of human chromoblastomycosis (*1**–**3*), a chronic cutaneous and subcutaneous infection characterized by slowly expanding nodules that eventually lead to emerging, cauliflower-like, mutilating and disfiguring eruptions. Infection proceeds with muriform cells in tissue provoking a granulomatous immune response. In areas where it is endemic, disease incidence is high. Yegres et al. (*4*) and Yëgues-Rodriguez et al. (*5*) noted a frequency of 16 cases/1,000 population under arid climatic conditions in rural communities of Venezuela; chromoblastomycosis in that region is caused mainly by *Cladophialophora carrionii*. In contrast, *Fonsecaea* spp. are prevalent in humid tropical climates. Esterre et al. (*6*) reported 1,343 cases of chromoblastomycosis from Madagascar, 61.8% of which were caused by *Fonsecaea* spp. Kombila et al. (*7*) reported 64 cases in Gabon (equatorial Africa), all caused by *Fonsecaea* spp., and Silva et al. (*8*) cited 325 cases in the Amazon region of Brazil, 98% of which had *Fonsecaea* spp. as the etiologic agent. In Sri Lanka, 94% of 71 chromoblastomycosis cases were caused by *Fonsecaea* spp (*9*).

*Fonsecaea* contains anamorphic ascomycetes belonging to the family *Herpotrichiellaceae* (order *Chaetothyriales*), which includes black yeasts and relatives (*10**–**12*). The genus comprises 3 sibling species: *F. pedrosoi*, *F. monophora*, and *F. nubica,* each of which has pathogenic potential (*10**,**13**,**14*). Infection process and routes of dispersal are insufficiently clarified. Humans presumably acquire the infection after being pricked by contaminated thorns or wood splinters, but some agents are substantially more clinically prevalent than their predominantly (hitherto unnamed) environmental counterparts (*15*), which indicates that infection is not a random process. In many published case reports, etiologic agents were referred to as *Phialophora pedrosoi* or identified with the obsolete name *F. compacta*, now known to be a mutant *F. pedrosoi* (*9**,**13**,**16*). Strains are no longer accessible for molecular verification. Hence, no data are available on the epidemiology of the species as defined by sequence data.

Phylogenetically, *Fonsecaea* spp. agents of chromoblastomycosis are flanked by nonpathogenic species (*10*) growing on plant debris. Discovery of natural habitat and source of infection by entities emerging on the human host is essential for understanding the evolution of pathogenicity. We present an amplified fragment-length polymorphism (AFLP) DNA fingerprinting study of a worldwide collection of clinical isolates that were identified as *Fonsecaea* spp. by state-of-the-art sequencing methods, supplemented with environmental isolates of the same species. The AFLP technique is a powerful method for discrimination between fungal species and for providing high-resolution fingerprinting data within species (*17**–**19*).

## Materials and Methods

### Fungal Strains and Culture Conditions

We studied 81 isolates representing the 3 currently recognized *Fonsecaea* spp. Geographic origins and hosts of the strains are listed in the Table A1; the set include reference strains from the Centraalbureau voor Schimmelcultures (CBS-KNAW Fungal Biodiversity Centre, Utrecht, the Netherlands) and fresh isolates from patients and from the environment. Stock cultures were maintained on slants of 2% malt extract agar and oatmeal agar at 24°C.

### DNA Extraction and Identification

Approximately 1 cm^2^ of 14- to 21-day-old cultures were transferred to 2 mL Eppendorf tubes containing 400 µL TEx buffer (Sigma-Aldrich, Zwijndrecht, the Netherlands), pH 9.0 (100 mmol Tris, 40 mmol Na-EDTA) and glass beads (Sigma G9143, Sigma-Aldrich). The fungal material was homogenized with a MoBio vortex (Bohemia, New York, USA) for 1 min. Subsequently, 120 µL of a 10% sodium dodecyl sulfate solution and 10 µL proteinase K (10 mg/mL, Sigma-Aldrich) were added and incubated for 30 min at 55°C; the mixture was vortexed for 3 min. After addition of 120 µL of 5M NaCl and 1/10 vol 10% cetyltrimethylammonium bromide solution (Sigma-Aldrich), the material was incubated for 60 min at 55°C. Then the mixture was vortexed for 3 min. Subsequently, 700 µL SEVAG (24:1, chloroform: isoamyl alcohol) was added, mixed carefully, and centrifuged for 5 min at 4°C at 20,400 × *g*. The supernatant was transferred to a new Eppendorf tube with 225 µL 5M NH_4_ acetate (Sigma-Aldrich), mixed carefully by inverting, incubated for 30 min on ice water, and centrifuged again for 5 min at 4°C at 20,400 × *g*. The supernatant was then transferred to another Eppendorf tube with 0.55 vol isopropanol and centrifuged for 5 min at 20,400 × *g*. Finally, the pellet was washed with 1 mL ice cold 70% ethanol. After drying at room temperature, it was resuspended in 48.5 µL TE buffer (Sigma-Aldrich) (Tris 0.12% wt/vol, Na-EDTA 0.04% wt/vol) and 1.5 µL of RNase (Sigma-Aldrich) and incubated in 37°C for 20–30 min. Quality of genomic DNA was verified on agarose gel. Species were identified on the basis of internal transcribed spacer (ITS), partial cell division cycle (*CDC42*), β-tubulin (*BT2*), and ACT sequences (*10**–**14*).

### AFLP Fingerprinting

We followed a protocol provided by the manufacturer (Applied Biosystems, Nieuwerkerk aan de IJssel, the Netherlands), with some minor modifications (*20**–**23*). Analyses were performed with 100–200 ng DNA.

### Restriction and Ligation of Adaptors

Two μL of DNA (100 ng/μL) was added to 9 μL restriction and ligation mixture (1.1 μL T4 DNA ligase buffer [Applied Biosystems]), 1.1 μL M NaCl, 2 U *Mse*I endonuclease, 10 U *Eco*RI endonuclease (New England Biolabs, Ipswich, UK), 30 U T4 DNA ligase, 1 μL *Mse*I-adaptor, 1 μL *Eco*RI-adaptor, and 3 μL dH_2_0 and incubated at 37°C for 2.5 h. Subsequently, each restriction/ligation reaction was diluted ≈3× by adding 25 μL demineralized water.

### Preselective and Selective PCR

In preselective PCR, 2 μL of diluted restriction/ligation product was added to 7.5 μL of AFLP core mix (Applied Biosystems), 0.25 μL of the *Eco*RI core sequence (5′-GAC TGC GTA CCA ATTC-3′), and 0.25 μL of the *Mse*I core sequence (5′-GAT GAG TCC TGA GTAA-3′). The mixture was amplified in an iCycler (Bio-Rad, Hercules, CA, USA) under the following conditions: 2 min at 72°C, followed by 20 cycles of 20 s at 94°C, 30 s at 56°C, and 2 min at 72°C. Each preselective PCR was diluted 2× by adding 10 μL of dH_2_O. In selective PCR, 1.5 μL of diluted preselective PCR products was mixed with 8.5 selective PCR mix containing 0.5 μL *Eco*RI-AC (labeled with FAM [6-carboxy fluorescein]), 0.5 μL *Mse*I-A, and 7.5 μL AFLP core mix (Applied Biosystems). The selective PCR conditions were cycling for 2 min at 94°C, followed by 10 cycles of 20 s at 94°C and 30 s at 66°C (decreasing 1°C with each subsequent cycle), and a final extension of 2 min at 72°C. This sequence was followed by 25 cycles of 20 s at 94°C, 30 s at 56°C, and 2 min at 72°C, and a final incubation of 30 min at 60°C.

### AFLP Analysis

FAM-labeled products were prepared for analysis in an ABI PRISM 377 Genetic Analyzer (Applied Biosystems) as follows: the selective PCR products were cleaned with Sephadex G-50, and selective PCR products were mixed with LIZ 500 in the new plate by several times pipetting (first by preparing master mix [8.7 µL demineralized water plus 0.3 µL Liz 500], then mixing this with 1.0 µL of selective PCR product by pipetting). The total volume was adjusted to 10 µL with dH_2_O. Denaturation was done at 95°C for 5 min, and then the reaction was snap-cooled on ice water. The LIZ 500 internal size standard in each sample was used for normalization of the fingerprint pattern according to the instruction manual. The densitometric curves were analyzed with BioNumerics software package (version 4.61, Applied Maths, Kortrijk, Belgium), by using the cosine similarity coefficient and the unweighted pair group method with arithmetic means cluster analysis. Statistical reliability of the cluster was investigated by using a cophenetic value, which calculates the correlation between the calculated and the dendrogram-derived similarity. Subdivisions in clusters were checked visually if they were supported by the banding patterns.

## Results

**Figure A1:**
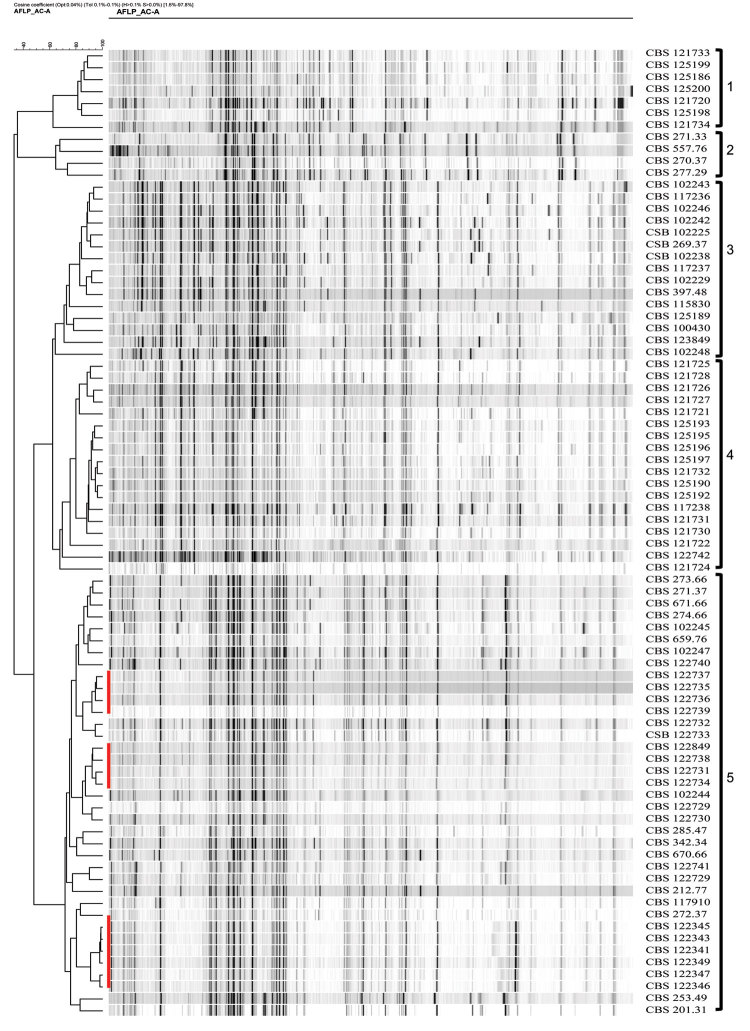
Clustering of amplified fragment-length polymorphism banding pattern of isolates of *Fonsecaea* spp. analyzed by using unweighted pair group method with arithmetic means. Red bars indicate clonal dispersal. Clusters 1 and 2 are *F. nubica*, clusters 3 and 4 are *F. monophora*, and cluster 5 is *F. pedrosoi*.

Profiles of 81 strains were generated with the *Eco*RI-AC + *Mse*I-A PCR adaptors. Fingerprints contained ≈60–70 bands in a 50–500-bp range. Another selective PCR with *Eco*RI core sequence+C and *Mse*I core sequence+A primer combination used elsewhere in related fungi (*24*) resulted in nonscorable fingerprints because of amplification of too many or only faint bands. Dendrograms derived from the AFLP banding patterns of *Fonsecaea* spp. were generated by using the unweighted pair group method with arithmetic means cluster analysis (Figure A1). At >62.50% similarity, 3 main clusters were found that matched with existing species on the basis of multilocus sequence analysis (ITS, *CDC42*, *BT2,* and *ACT1*), i.e., *F. pedrosoi*, *F. monophora*, and *F. nubica*. At an automatic cutoff value option set at <62.5% similarity, the *F. monophora* and *F. nubica* clusters were subdivided in 2 evident groups each, leading to a total of 5 clusters (1–5) interpreted as populations. Clusters 1 and 2 matched with *F. nubica*, clusters 3 and 4 with *F. monophora*, and cluster 5 with *F. pedrosoi*. Individual bands varied within the profiles, but further subclustering was limited, e.g., in a slightly deviating derived subclade in population 5. The groups defined above by AFLP analysis are interpreted as populations (1–5) in the text below. In population 5, some strains were nearly 100% identical, e.g., CBS 122341, 122343, 122345, and 122349, all originating from patients with chromoblastomycosis in Mexico City, Mexico (Figure A1; Table A1).

We determined the geographic distributions of the 5 main populations of *Fonsecaea* strains (Figure). Areas endemic for *Fonsecaea*, judging from the literature, are in tropical and subtropical climate zones. Population 1 comprised a cluster of *F. nubica* strains originating from humans with chromoblastomycosis in Guangdong, People’s Republic of China. Population 2 of the same species comprised 4 strains, 2 of which originated from humans with chromoblastomycosis in South America, 1 from France, and 1 with unknown origin. The profiles were too different to trace to any clonal identity. Population 3 (*F. monophora*) comprised 15 strains, most of which were isolated from humans with chromoblastomycosis in South America; 1 originated from the United States, and 1 originated from Haikou in southern China. Two strains were isolated from decaying plants in Brazil, and the second US strain was derived from a human with a brain infection. Two other strains from human brain infections in Brazil and in Africa had unique profiles that could not be unambiguously linked to any other isolate. Another African strain, from a patient with chromoblastomycosis who lived in Spain and had acquired the infection 36 years earlier in Guinea (*25*), also had a unique profile. Population 4 of *F. monophora* comprised 16 strains from Guangdong in southern China, and 1 came from Shandong, ≈1,850 km distant. All had derived from humans with chromoblastomycosis. A single sample originated from a patient with a brain infection who lived in the United Kingdom (*26*); whether the patient had visited southern China could not be established. In population 5 (*F. pedrosoi*), most strains originated from chromoblastomycosis patients in Central and South America. Some geographic clustering was visible, i.e., the derived group of strains from South America (uppermost clade of population 5 in the Figure A1) was segregated from those from Central America. Several of the strains from South American originated from soil and were isolated through mouse passage. One strain from an ear of a gazelle in Libya and 1 from a human with chromoblastomycosis in the Netherlands could not directly be linked to any other strain.

## Discussion

AFLP typing is comparable to use of other DNA markers, such as random amplified polymorphic DNA, restriction fragment-length polymorphism, or microsatellites, in terms of time and cost efficiency, reproducibility, and resolution (*27*). The technique has emerged as a major epidemiologic tool with broad application in ecology, population genetics, pathotyping, DNA fingerprinting, and quantitative trait loci mapping (*28*). AFLP fingerprinting is useful for the molecular characterization of microorganisms with relatively large genomes, including various fungal species (*18**,**19**,**21**–**23**,**29**,**30*). In a preliminary experiment that used different primer combinations, the combination *Eco*RI-AC + *Mse*I-A adaptors gave excellent results, yielding readable profiles with well-separated bands.

The degree of variation in *Fonsecaea* appeared to differ between species. The major 5 clusters were separated at <62.5% similarity, with significant differences in the presence of major fragments, several of which were unique to individual isolates or subpopulations. Populations 1 and 2, 3 and 4, and 5 corresponded with species borderlines established recently by Najafzadeh et al. (*10**,**14*) on the basis of multilocus sequencing with ITS, *CDC42*, *BT2*, and *ACT1*. Population 5 (*F. pedrosoi*) varied least at >71.7% similarity, with limited reproducible substructure being discernable. Nearly all isolates of this species originated from South and Central America (Venezuela, Brazil, Mexico, Argentina, Puerto Rico, and Uruguay). One isolate from a human with chromoblastomycosis in the Netherlands was likely to have been imported (*13*). One isolate from a gazelle ear in Libya, northern Africa, was the only geographic exception that could not be explained. Clusters of strains that could be grouped as being visually identical and with similarities >71.7% (Figure A1; Table A1) were mostly collected at close geographic distance from each other. This finding suggests that vectors of dispersal for *Fonsecaea* spp. are slow, leading to detectable regional diversification. The relatively low degree of variation of *F. pedrosoi* and confinement to Central and South America indicate a founder effect, the species being the most recently emerged taxon in *Fonsecaea*. *F. monophora* and *F. nubica* were distributed worldwide but were geographically diverse in that population 4 of *F. monophora* was nearly confined to China, with highly similar profiles (Figure A1). One strain of this population 4, CBS 117238, originated from a brain infection in a human in the United Kingdom; whether this patient had emigrated from China could not be determined from the original publication (*25*). *F. monophora* population 3 was found mainly in the Western Hemisphere, particularly in Brazil. Judging from the near identity of profiles of strains isolated in 1937 (CBS 271.37) and in 1999 (CBS 102245) (Figure A1), we can conclude that clones are maintained locally over decades. The 2 US strains presumably derived from immigrants from South America or Central America. Population 3 was also found in Africa and in Haikou in China, 600 km from Guangdong, where population 4 of *F. monophora* is prevalent. Strains of *F. nubica* show a similar bipartition over Asia and the Western Hemisphere, with a prevalently Chinese (population 1) and a prevalently Brazilian (population 2) population, and a presumed infected immigrant in France. Kawasaki et al. (*31**,**32*) provided similar data on the basis of restriction fragment-length polymorphism of mitochondrial DNA, showing that *Fonsecaea* spp. from Japan and China differed consistently from isolates from Central and South America.

Nearly all *Fonsecaea* spp. isolates available in culture collections originate from mammals, mostly humans with chromoblastomycosis, and were rarely recovered from the environment of symptomatic patients despite several attempts (*33*). Occasionally, *F. pedrosoi* was isolated from mice that were euthanized for isolation of black yeasts after they had been inoculated with environmental samples (*34*). This information suggests that *Fonsecaea* spp., particularly *F. pedrosoi*, have a competitive advantage by using this enrichment source. Mouse passage proved to be more efficient for environmental isolation of etiologic agents of chromoblastomycosis than general methods such as oil flotation (*35*). The latter technique mostly isolates other environmental *Fonsecaea* spp. that are not known to be pathogenic to humans (*33*).

In humans with chromoblastomycosis, the male:female ratio of patients is 63:2. This male preponderance of 97% cannot be explained by different exposition rates. Distinct male preponderance is also noted in the neurotropic relative, *Cladophialophora bantiana* (G.S. de Hoog, unpub. data). Population 3 of *F. monophora* has a wider clinical spectrum than the remaining groups, comprising, in addition to chromoblastomycosis, several isolates from human brain infection. This population also comprised some isolates from soil and plant debris acquired without use of mammal baits. Coexistence of closely interrelated entities differing in pathogenicity and virulence seems likely in *Fonsecaea* spp., as was also suggested for black yeasts (A.H.G. Gerrits van den Ende et al., unpub. data).

Our data demonstrate that AFLP fingerprinting is a tool that produces highly reproducible results for molecular epidemiology. The use of AFLP showed that local *Fonsecaea* agents of chromoblastomycosis seem able to be maintained over 70 years, and therefore epidemiologic profiles take the structure of expanding clones. By locality, patients are infected by only a limited number of genotypes. The fungi disperse slowly, leading to appreciable geographic structuring, which ultimately may lead to allopatric speciation (diversification resulting from geographic barriers). Few environmental strains have been recovered during repeated isolation experiments, whereas *Fonsecaea* spp. accumulates substantially in the human host. The mechanisms behind their pathology remain unexplained.

## Figures and Tables

**Figure:**
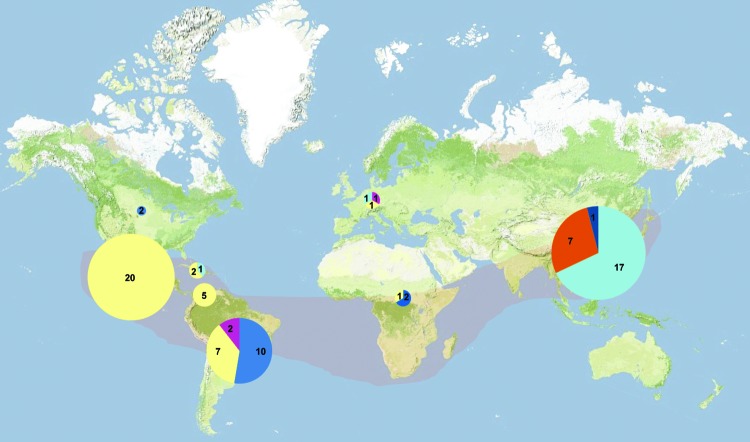
Geographic distribution of *Fonsecaea* spp. samples analyzed by using amplified fragment-length polymorphism. Light pink shading indicates zone of clinical *Fonsecaea* spp. endemicity, according to published case reports. Sizes of pies and numbers reported within the pies denote the number of strains examined; colors represent *Fonsecaea* spp. populations: orange, *F. nubica* population 1; fuchsia, *F. nubica* population 2; dark blue, *F. monophora* population 3; light blue, *F. monophora* population 4; yellow, *F. pedrosoi* population 5.

## Appendix

**Table A1:** *Fonsecaea* spp. isolates used for amplified fragment-length polymorphism analyses

Taxonomic name	CBS number	Other reference(s)	Origin	Host/sex	Location	Population
*F. nubica*	CBS 121733	dH 18411, SUMS 0011	Chromoblastomycosis	Human/M	China, Guangdong	1
	CBS 125199	dH 20427	Chromoblastomycosis	Human/F	China, Guangdong	1
	CBS 125186	dH 20429	Chromoblastomycosis	Human/M	China, Guangdong	1
	CBS 125200	dH 20425	Chromoblastomycosis	Human/M	China, Guangdong	1
	CBS 121720	dH 18398, SUMS 0251	Chromoblastomycosis	Human/M	China, Guangdong	1
	CBS 125198	dH 20418	Chromoblastomycosis	Human/M	China, Guangdong	1
	CBS 121734	dH 18412, SUMS 0255	Chromoblastomycosis	Human/M	China, Guangdong	1
	CBS 271.33	dH 15659, ATCC 18658, IMI 134458	Chromoblastomycosis	Human/M	South America	2
	CBS 557.76	ATCC 28174	Unknown	Unknown	Unknown	2
	CBS 270.37	dH 15657	Unknown	Unknown	France	2
	CBS 277.29	dH 15668	Chromoblastomycosis	Human/M	Brazil	2
*F. monophora*	CBS 102243	dH 11607	Chromoblastomycosis	Human/M	Brazil, Parana, Ibituva	3
	CBS 117236	dH 15330, UTHSC 04-2904	Brain	Human/M	United States	3
	CBS 102246	dH 11611	Chromoblastomycosis	Human/M	Brazil, Parana, Campo Largo	3
	CBS 102242	dH 11606	Chromoblastomycosis	Human/M	Brazil, Santa Catarina, Curitibanos	3
	CSB 102225	dH 11585	Decaying wood	Plant	Brazil, Parana, Colombo	3
	CSB 269.37	dH 12659	Chromoblastomycosis	Human	South America	3
	CSB 102238	dH 11602	Soil	Soil	Brazil, Parana, Tibagi River	3
	CBS 117237	dH 15331, UTHSC 04-2631	Chromoblastomycosis	Human/M	United States	3
	CBS 102229	dH 11590	Decaying vegetable cover	Plant	Brazil, Parana, Piraquara	3
	CBS 397.48	dH 15828, ATCC 9475	Chromoblastomycosis	Human/M	South America	3
	CBS 115830	dH 12978	Brain	Human/M	Brazil	3
	CBS125189	dH 20421	Chromoblastomycosis	Human/M	China, Haikou	3
	CBS 100430	ATCC 32280	Brain	Human/M	Africa	3
	CBS 123849	dH 20215	Chromoblastomycosis	Human/F	Africa, Guinea	3
	CBS 102248	dH 11613	Chromoblastomycosis	Human/M	Brazil, Parana, Piraquara	3
	CBS 121725	dH 18403, SUMS 0250	Chromoblastomycosis	Human/M	China, Guangdong	4
	CBS 121728	dH 18406, SUMS 0158	Chromoblastomycosis	Human/M	China, Guangdong	4
	CBS 121726	dH 18404, SUMS 0192	Chromoblastomycosis	Human/M	China, Guangdong	4
	CBS 121727	dH 18405, SUMS 0190	Chromoblastomycosis	Human/M	China, Guangdong	4
	CBS 121721	dH 18399, SUMS 0246	Chromoblastomycosis	Human/M	China, Guangdong	4
	CBS 125193	dH 20426	Chromoblastomycosis	Human/M	China, Guangdong	4
	CBS 125195	dH 20417	Chromoblastomycosis	Human/M	China, Guangdong	4
	CBS 125196	dH 20419	Chromoblastomycosis	Human/M	China, Guangdong	4
	CBS 125197	dH 20420	Chromoblastomycosis	Human/M	China, Guangdong	4
	CBS 121732	dH 18410, SUMS 0012	Chromoblastomycosis	Human/M	China, Guangdong	4
	CBS 125190	dH 20422	Chromoblastomycosis	Human/M	China, Guangdong	4
	CBS 125192	dH 20424	Chromoblastomycosis	Human/M	China, Guangdong	4
	CBS 117238	dH 13130, UTHSC R-3486	Brain	Human	United Kingdom, England	4
	CBS 121731	dH 18409, SUMS 0013	Chromoblastomycosis	Human/M	China, Guangdong	4
	CBS 121730	dH 18408, SUMS 0014	Chromoblastomycosis	Human/M	China, Guangdong	4
	CBS 121722	dH 18400, SUMS 0247	Chromoblastomycosis	Human/M	China, Guangdong	4
	CBS 122742	dH 19251, SUMS 0147	Chromoblastomycosis	Human	China, Shandong	4
	CBS 121724	dH 18402, SUMS 0200	Chromoblastomycosis	Human/M	China, Guangdong	4
*F. pedrosoi*	CBS 273.66	dH 15663	Mouse passage	Soil	Venezuela	5
	CBS 271.37	dH 15659, ATCC 18658, IMI 134458	Chromoblastomycosis	Human/M	South America	5
	CBS 671.66	dH 16159	Mouse passage	Soil	Venezuela	5
	CBS 274.66	dH 15665	Mouse passage	Soil	Venezuela	5
	CBS 102245	dH 11610	Chromoblastomycosis	Human/M	Brazil, Parana, Ampere	5
	CBS 659.76	dH 16142, ATCC 28303	Chromoblastomycosis	Human/M	Argentina	5
	CBS 102247	dH 11612	Chromoblastomycosis	Human/M	Brazil, Parana	5
	CBS 122740	dH 18430, Bonifaz 002200	Chromoblastomycosis	Human/M	Mexico, Mexico City	5
	CBS 122737	dH 18896	Chromoblastomycosis	Human/M	Mexico, Mexico City	5a
	CBS 122735	dH 18898	Chromoblastomycosis	Human/M	Mexico, Mexico City	5a
	CBS 122736	dH 18897	Chromoblastomycosis	Human/M	Mexico, Mexico City	5a
	CBS 122739	dH 18894	Chromoblastomycosis	Human/M	Mexico, Mexico City	5a
	CBS 122732	dH 18901	Chromoblastomycosis	Human/M	Mexico, Mexico City	5
	CSB 122733	dH 18900	Chromoblastomycosis	Human/M	Mexico, Mexico City	5
	CBS 122849	dH 18902	Chromoblastomycosis	Human/M	Mexico, Mexico City	5b
	CBS 122738	dH 18895	Chromoblastomycosis	Human/M	Mexico, Mexico City	5b
	CBS 122731	dH 18903	Chromoblastomycosis	Human/M	Mexico, Mexico City	5b
	CBS 122734	dH 18899	Chromoblastomycosis	Human/M	Mexico, Mexico City	5b
	CBS 102244	dH 11608	Chromoblastomycosis	Human/M	Brazil, Parana, Ipora	5
	CBS 122729	dH 18905	Chromoblastomycosis	Human/M	Mexico, Mexico City	5
	CBS 122730	dH 18904	Chromoblastomycosis	Human/M	Mexico, Mexico City	5
	CBS 285.47	dH 15680, ATCC 10222	Chromoblastomycosis	Human/M	Puerto Rico	5
	CBS 342.34	dH 15773	Chromoblastomycosis	Human/M	Puerto Rico	5
	CBS 670.66	dH 16157	Mouse passage	Soil	Venezuela	5
	CBS 122741	dH 18431, Bonifaz 02300	Chromoblastomycosis	Human/M	Mexico, Mexico City	5
	CBS 122729	dH 18905	Chromoblastomycosis	Human	Mexico, Mexico City	5
	CBS 212.77	dH 15549	Chromoblastomycosis	Human/M	Netherlands, Amsterdam	5
	CBS 117910	dH 14477	Chromoblastomycosis	Human/M	Venezuela, Coro, Falcón State	5
	CBS 272.37	dH 15661	Chromoblastomycosis	Human	Brazil	5
	CBS 122345	dH 18914, Bonifaz 121-06	Chromoblastomycosis	Human/M	Mexico, Mexico City	5c
	CBS 122343	dH 18916, Bonifaz 122-07	Chromoblastomycosis	Human/M	Mexico, Mexico City	5c
	CBS 122341	dH 18918, Bonifaz 345-07	Chromoblastomycosis	Human/M	Mexico, Mexico City	5c
	CBS 122349	dH 18910, Bonifaz 234-04	Chromoblastomycosis	Human/M	Mexico, Mexico City	5c
	CBS 122347	dH 18912, Bonifaz 0257-05	Chromoblastomycosis	Human/M	Mexico, Mexico City	5c
	CBS 122346	dH 18913, Bonifaz 333-05	Chromoblastomycosis	Human/M	Mexico, Mexico City	5c
	CBS 253.49	dH 15620	Chromoblastomycosis	Human	Uruguay, Montevideo	5
	CBS 201.31	dH 15523	Gazelle, ear	Animal	Libya, Cyrenaica, Derna	5

*ATCC, American Type Culture Collection, Manassas, VA, USA; CBS, Centraalbureau voor Schimmelcultures, Utrecht, the Netherlands; dH, G.S. de Hoog working collection; SUMS, Sun Yat-Sen University Medical Science, Guangzhou, People’s Republic of China; IMI, International Mycological Institute, London, UK; UTHSC, Fungus Testing Laboratory, Department of Pathology, University of Texas Health Science Center at San Antonio, San Antonio, TX, USA.
